# Online Cognitive Behavioral Therapy (CBT) Life Skills Program for Depression: Pilot Randomized Controlled Trial

**DOI:** 10.2196/30489

**Published:** 2022-02-17

**Authors:** Christopher Williams, Carrie-Anne McClay, Rebeca Martinez, Jill Morrison, Caroline Haig, Ray Jones, Paul Farrand

**Affiliations:** 1 Institute of Health and Wellbeing University of Glasgow Glasgow United Kingdom; 2 Mersey Care National Health Service Foundation Trust Norris Green Community Hub Liverpool United Kingdom; 3 Faculty of Health University of Plymouth Plymouth United Kingdom; 4 Sir Henry Wellcome Building for Mood Disorders Research University of Exeter Exeter United Kingdom

**Keywords:** pilot study, depression, low mood, iCBT, guided self-help, online, psychotherapy, LLTTF, RCT, treatment gap, bibliotherapy, life skills, anxiety

## Abstract

**Background:**

Depression is a common mental health problem with significant personal and social consequences. Studies have suggested that cognitive behavioral therapy (CBT) is an effective treatment for depression and anxiety when delivered one-to-one by an expert practitioner, but access to this talking therapy is often limited, and waiting lists can be long. However, a range of low-intensity interventions that can increase access to services are available including guided CBT self-help materials delivered via books, classes, and online packages.

**Objective:**

This project aimed to pilot a randomized controlled trial (RCT) of an online CBT-based life skills course with community-based individuals experiencing depression.

**Methods:**

Individuals with symptoms of depression were recruited directly from the community via newspaper advertisements. Participants were remotely randomized to receive either immediate access (IA) or delayed access (DA) to a research version of the Living Life to the Full online CBT-based life skills package (3rd edition) with telephone support provided by nonspecialist, charity-based workers while they used the online intervention. The primary end point was at 3 months postrandomization, at which point, the DA group were offered the intervention. Levels of depression, anxiety, social functioning, and satisfaction were assessed.

**Results:**

There were effective recruitment, randomization, and uptake, with 19 IA and 17 DA control participants entering the pilot study via newspaper advertisements and 13 of the 19 participants taking up the intervention. Overall, 72% (26/36) were not currently under the care of their general practitioner. The online package was acceptable to participants; the mean satisfaction score on the Client Satisfaction Questionnaire was 21 out of 32 (SD 8.89). At 3 months, data collection was achieved from 78% (28/36) of the participants. The efficacy and retention data were used for a power calculation indicating that 72 participants in total will be required for a future substantive RCT.

**Conclusions:**

The research design successfully tested the recruitment, data collection, and intervention delivery. The pilot study has provided data for the required sample size for the full RCT.

**Trial Registration:**

ISRCTN registry ISRCTN12890709; https://doi.org/10.1186/ISRCTN12890709

**International Registered Report Identifier (IRRID):**

RR2-10.1186/s13063-016-1336-y

## Introduction

### Background

Depression is characterized by persistent low mood and loss of pleasure or interest in things. Additional symptoms include sleep problems, loss of energy, changes in appetite, trouble concentrating, feeling like a failure, and thoughts of self-harm or suicide [1]. These symptoms can have a significant impact on social functioning, physical health, work, and relationships and can result in suicide. The most recent UK Wellbeing Survey found that 19.1% of people aged 16 years and older in the United Kingdom show evidence of anxiety and depression [2].

In general practice, symptoms of depression are usually assessed using the Patient Health Questionnaire (PHQ) [3] or similar self-report scale. Following this, there are various treatments that can be recommended. The National Institute for Health and Clinical Excellence (NICE) [4] guidelines state that treatments for depression should follow the stepped care model, taking into account severity and chronicity of symptoms and response to treatment. Treatment options range from active monitoring and psychoeducation at step 1 to psychosocial treatment options such as low-intensity interventions including bibliotherapy (cognitive behavioral therapy [CBT]–informed self-help books), internet-based cognitive behavioral therapy (iCBT), and group CBT within step 2; Step 3 involves face-to-face delivery of therapy such as specialist-delivered CBT, interpersonal therapy, and behavioral activation; and the use of crisis and inpatient services represent steps 4 and 5.

However, the majority of individuals with depression worldwide do not receive appropriate treatment for their depression within health services [5]. This treatment gap may be dependent upon a number of factors, including nonrecognition of the problem (by the individual or health care workers), reluctance to seek treatment from statutory mental or health service provision, limited knowledge about treatment options, or a lack of mental health resources resulting in long waiting lists. A key aim of the World Health Organization mental health Gap Action Programme (mhGAP) is to increase access to psychological therapies via nonexpert support or health care workers. This could be achieved through the application of low-intensity CBT-guided self-help interventions, delivered via bibliotherapy or online in the form of iCBT. These could be delivered, as recommended, in Step 2 of the stepped care model and through enhancing “informal health care” service provision through trained members of community or voluntary sector organizations [6] to improve access for those reluctant to present to health services.

Various iCBT packages are available, including MoodGYM [7], Beating the Blues [8], and Living Life to the Full (LLTTF) [9]. There is evidence that online CBT can be an acceptable, impactful, and cost-effective intervention for depression [10,11] Some studies have found iCBT to be as effective as face-to-face CBT [12,13].

A review carried out by Richards and Richardson [14] found that outcomes were best when iCBT interventions were delivered with some form of support rather than unguided. This review highlighted that drop-out rates are high in studies of iCBT (57% across the 40 studies). The issues of support, guidance, and retention are key in interpreting the “REEACT” study of iCBT for depression by Gilbody et al [15]. This study found no significant effect of free (MoodGYM) or commercial (Beating the Blues) iCBT packages when used with purely technical support (as opposed to clinical support) at 4 months compared with usual general practitioner (GP) care. However, significant methodological concerns have been highlighted concerning the support offered, with caution expressed regarding the conclusion reached [16]. Poor rates of uptake were experienced alongside nearly one-quarter (24%) of participants dropping out by 4 months. Furthermore, those engaging with the intervention only completed 1 to 2 modules with just over 6 minutes in total of technical telephone support received on average. Such small amounts of support are below that recommended for so-called minimal contact support [17]. Furthermore, the support was described as technical support alone, which means that the results need to be interpreted with caution. In contrast, support for iCBT interventions is usually provided by a therapist or trained facilitator and improves outcomes [14]. Such content-focused support usually involves motivating users, goal setting, and review of homework tasks, with the main focus being on working through the online package and applying what is learned.

This study is a pilot study of an online educational life skills program designed for low mood and anxiety based on CBT principles. The LLTTF approach puts bibliotherapy and life skills training at its core and adopts a blended learning approach [18] where the user engages with the course through a variety of media (reading, audiovisual modules, and video). Content addresses key aspects of low mood and stress and is available in different formats to suit the user’s preferred learning style. This includes live classes [19-21], printed books [19], and electronic books in addition to the online course. The online version of the LLTTF intervention adheres to the key NICE recommendations regarding iCBT packages in terms of content, number of modules, and the support element provided [4]. However the person chooses to learn, it is recommended that a clear and structured support protocol is offered to enhance package engagement and application. A nonrandomized feasibility study [22] demonstrated equivalent outcomes for the online course [9] compared with the Beating the Blues online resource [8] and a book-based bibliotherapy intervention [23,24]. However, as there are currently no adequately powered randomized controlled trials (RCTs) of the LLTTF package, further research is needed to examine the efficacy of the package in its online format.

### Research Questions

#### Primary Question Examining Methodological Uncertainties

The primary research question was: “Is the study design feasible—is it possible to recruit from the community, randomize participants, deliver the online intervention with structured telephone support, and collect data at baseline and 3 months post randomization?”

#### Secondary Questions Examining Adherence and Acceptability

The secondary research questions were: (1) “To what extent will participants adhere to the online intervention?” (2) “Is the Living Life to the Full online package acceptable to participants?” and (3) “What sample size will be required in the future substantive study?”

## Methods

### Overview

The study was a parallel, 2-arm, pilot RCT with a 50:50 allocation ratio across the 2 groups with the primary outcome point at 3 months postrandomization. Participants were remotely randomized to receive the online intervention immediately (IA group) or following a 3-month delay after randomization (DA group). The DA group served as the control at the primary outcome point.

### Ethics Approval

Ethical approval was granted by the College of Medical, Veterinary and Life Sciences Ethics Committee for Non-Clinical Research Involving Human Subjects, University of Glasgow (Reference number 200140159).

### Procedures

#### Recruitment and Participants

The aim was to recruit 30 to 50 participants, a sample size widely accepted as sufficient for pilot studies to identify problems with recruitment, delivery of the intervention, and evaluation measures [25]. People with symptoms of depression were recruited using a community-based approach using advertisements in the Metro free newspaper in Central Scotland. No participants were recruited directly via the National Health System (NHS). Informed consent was collected from all potential participants prior to entry into the study.

#### Inclusion Criteria

Participants had to meet the following criteria: age ≥18 years; living in the United Kingdom; able to understand written and spoken English; regular access to a computer, smartphone, and tablet with audio and broadband connections; and PHQ-9 score ≥10 [3].

#### Exclusion Criteria

Participants were excluded for any of the following reasons: high rating for suicidality (ie, scoring 2 or 3 on PHQ-9 item 9), currently receiving any psychological intervention such as counselling or psychotherapy, new or altered dose of antidepressant in the last month, or taking part in any other research projects.

#### Randomization

Participants completed initial online questionnaires, and those that were eligible and consented were then remotely randomized to either the IA or DA group. Participants’ ID numbers were passed to a separate researcher who used the randomization function in Excel to remotely assign participants to the immediate IA or DA group. As it was a pilot study, we did not stratify for any variables during randomization. After randomization, those in the IA group were given a free access code and link to the LLTTF website, including instructions for how to use the resources. Those in the DA group were told that they would be contacted again in 3 months; the free access code to the LLTTF website was sent once follow-up data had been received.

### Intervention

The free-access, online LLTTF course consists of 8 modules that teach a range of CBT-based life skills.

Session 1 involves an introduction to the CBT model and the “vicious cycle” of low mood that aims to help participants understand why they feel the way they do and how their thoughts, feelings, physical symptoms, and behavior are linked.

The session 2 module focuses on the impact of reduced activity. Users are encouraged to consider the things that they have stopped or reduced doing as a result of their low mood and make a plan to re-establish these activities in order to improve their mood.

Session 3 teaches skills to help tackle upsetting thinking, including how to label unhelpful thoughts, identify negative thinking patterns, and turn these thoughts around to create more helpful ones.

Session 4 teaches how self-confidence is developed and teaches confidence-building techniques.

Session 5 involves a problem-solving approach using an “easy 4-step plan” to help break down problems into smaller parts in order to overcome challenges.

Session 6 addresses unhelpful behaviors that may be worsening mood. Participants are encouraged to recognize problem behaviors such as isolating themselves or drinking or smoking too much and then create a plan for reducing them.

In session 7, participants learn to recognize the things that cause them to feel irritable or angry and the early warning signs they experience when they start to feel angry. They then learn techniques for better managing their anger to help them react differently to challenging people and situations.

Session 8, which is the final module, teaches key lifestyle choices that can improve mood, including healthy eating, exercise, and closeness with others.

Additional course worksheets, video, and audio (relaxation) resources supplement the main modules. These are available on the intervention website.

### Support

The NICE guidelines [4] for the treatment of adults with depression advise that self-help packages should have support that includes monitoring and reviewing progress. This study included automated weekly email reminders plus 6 weekly support sessions provided over the telephone with a support worker. The support workers were volunteers from the Scottish depression charity Action on Depression who had completed at least one day of training with the Five Areas team (developers of LLTTF) and had experience with delivering LLTTF content and support. Support was protocol-driven and focused on encouraging use and application of the intervention [18]. It was designed to encourage an individualized plan to be made at the end of each session, completing Planner and Review worksheets informed by a “Plan, Do, Review” structure. Within this structure, participants apply the skills and techniques they have learned to make changes in their lives, using the materials to support them in doing this. The amount of support time allocated varied to reflect the complexity of the needs of the person being supported; session durations were not recorded in this pilot study although the recommended duration was 15 minutes to 20 minutes per session.

### Data Collection

Baseline measures included age, gender, ethnicity, mood, social function, and past and current sources of support. After 3 months, all randomized participants were invited to repeat mood and social functioning questionnaires. Use of the intervention and satisfaction with the online package in the IA group were assessed. The collection of data for an economic analysis was piloted at this time point. This involved completion of the Client Service Receipt Inventory (CSRI) [26] and EQ5D [27], which assess access to health care services and health-related quality of life. These data were not analyzed, and the purpose was to test the ability to collect the data.

### Outcome Measures

The key outcomes of the study were the ability to recruit and retain participants, to test the acceptability of the intervention as measured by the Client Satisfaction Questionnaire-8 (CSQ-8) [28], and the ability to gather data.

The measures used to assess the impact on mood and social functioning were the PHQ-9 [3], the Generalized Anxiety Disorder (GAD-7) questionnaire [29], and the Work and Social Adjustment Scale (WSAS) [30]. These were sent via a SurveyMonkey [31] link at each time point.

### Statistical Analyses

Recruitment, uptake, and adherence to the intervention are summarized. Baseline characteristics are summarized for all participants as well as for the IA and DA groups separately. Two-sample *t* tests or Mann-Whitney *U* tests, depending on distributions, and Fisher exact tests were used to compare the 2 treatment groups and confirm that participants had been effectively randomized.

To practice the analysis for efficacy, appropriate linear models were applied using an intention-to-treat approach. Models were adjusted using baseline scores as covariates. These models were used to estimate and test the statistical significance of the within-group change between baseline and 3 months and the between-group differences at each time point.

## Results

### Recruitment

The community-based recruitment method we employed was successful as our sample size target to recruit 30 to 50 participants was met within the proposed recruitment period (August 2015 to May 2016; see Table 1). Recruitment involved 3 distinct recruitment phases of advertising, each including 1 Metro newspaper advertisement appearance per week for 4 weeks. Therefore, the total number of Metro advertisements placed was 12.

**Figure 1:**
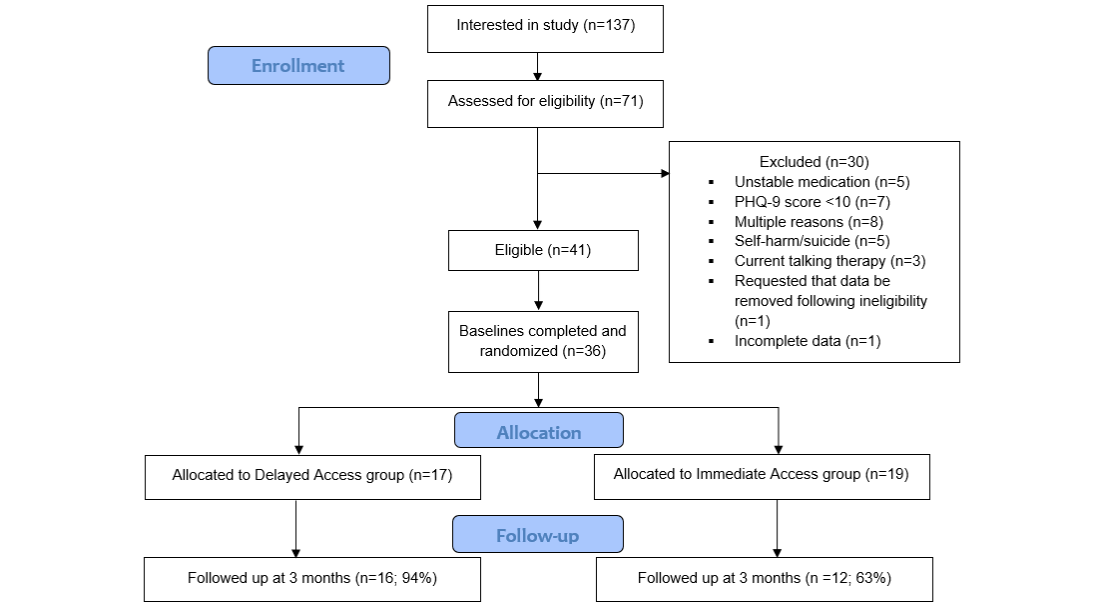
Study flowchart. PHQ-9: Patient Health Questionnaire-9.

Of the 70 potential participants who were assessed for eligibility, 36 individuals were randomized by the end of the recruitment period. As shown in the CONSORT flow diagram (Figure 1), 5 people were excluded due to thoughts of self-harm or suicide. These individuals were advised to contact their GP regarding these feelings and were also given a list of additional sources of support that they could access.

As outlined in Table 2, in the randomized sample, the mean chronicity of depressive symptoms was 9.20 (SD 8.70) years. Symptoms had persisted for at least 2 years for 78% (28/36) of participants and for at last 5 years for 56% (20/36), reflecting a chronic group. Despite this, 26 of the 36 participants (72%) were not currently under the care of their GP, and 20 of the 36 participants (56%) were getting no support at all for their low mood.

**Table 1:** Recruitment phases.

Recruitment phase	Dates	Number of advertisements
1	August 2015 to October 2015	4 (1 a week for 4 weeks)
2	January 2016 to March 2016	4 (1 a week for 4 weeks)
3	March 2016 to May 2016	4 (1 a week for 4 weeks)

**Table 2:** Demographic data.

Characteristic		Overall (n=36)	Control (n=17)	Intervention (n=19)
**Age (years)**				
	Mean (SD)	42.33 (13.54)	41.82 (15.38)	42.79 (12.09)
	Minimum to maximum	20.00 to 69.00	23.00 to 69.00	20.00 to 63.00
**Sex, n (%)**				
	Female	24 (67)	11 (65)	13 (68)
	Male	11 (31)	5 (29)	6 (32)
	Transgender	1 (3)	1 (6)	0 (0)
**Chronicity (years)^a^**				
	Mean (SD)	9.20 (8.70)	7.85 (6.54)	10.48 (10.39)
	Minimum to maximum	0.17 to 30.00	0.33 to 20.00	0.17 to 30.00
**Marital status, n (%)**				
	Married/cohabiting	17 (47)	8 (47)	9 (47)
	Unmarried/separated	19 (53)	9 (53)	10 (53)
**Antidepressant use, n (%)**				
	Yes	10 (28)	3 (18)	7 (37)
	No	26 (72)	14 (82)	12 (63)
**Ethnicity, n (%)**				
	White	36 (100)	17 (100)	19 (100)
	Non-White	0 (0)	0 (0)	0 (0)

^a^Because chronicity was not available for 1 person in the intervention group, the sample size for the overall group was 35, and the sample size for the intervention group was 18.

### Uptake and Adherence

These findings relate to the IA group participants only as the DA had not accessed the intervention at 3 months. Of the 19 IA participants, 13 provided data concerning the number of sessions they completed, and 10 (10/13, 77%) of the responding IA participants completed at least 1 module of the intervention. The average number of the 4 core modules completed was 2 (SD 1.58; minimum 0, maximum 4). One-third (4/13, 31%) of participants completed all 4 core modules. The mean number of optional modules completed was 1 (SD 1.41).

### Ability to Collect Data and Questionnaires

At the 3-month follow-up, primary outcome data were collected from 78% (28/36) of the participants. A Fisher exact test showed a significant between-group difference in follow-up rates (*P*=.03), with 94% (16/17) of the DA participants completing the measures at 3 months, compared with 63% (12/19) of the IA participants. Economic analysis data were collected from 25% (7/28) of responding participants at 3 months.

### Participant Satisfaction

Participant satisfaction with the LLTTF online package was measured using the CSQ-8 for the intervention group. Of the 19 IA participants, 10 completed this questionnaire. A mean satisfaction score of 21 (SD 8.89), from a possible score of 32, indicated that participants were reasonably satisfied with the online intervention and support they received.

In relation to side effects of the intervention, very few were reported. Specifically, 1 participant said that they felt bad about their ability to put what they learned in the course into action, 4 people felt that they were letting the support worker down during the intervention, and 1 person said that the course content made them feel worse.

### Initial Assessments of Efficacy

#### Effect on Depression (PHQ-9) Scores

The IA and DA groups improved to a similar level between baseline and 3 months. The mean reduction in PHQ-9 score in the IA group was 6.08 (SD 7.49); the mean difference in the DA group was 5.19 (SD 6.00). As expected in this small unpowered pilot study, the between-group difference was not statistically significant (*P*=.62; 95% CI –6.10 to 3.69).

#### Effect on Anxiety and Social Functioning

At 3 months, the IA group had improved by 4.60 (SD 6.53) points on the GAD-7 from baseline, while the average improvement in the DA group was 2.80 (SD 4.97) points. Again, no significant treatment effect was found for anxiety (*P*=.20; 95% CI –7.76 to 1.70). Similarly, there was no significant effect of treatment on social functioning (WSAS scores; *P*=.99, 95% CI –8.16 to 8.02).

## Discussion

### Principal Findings

This pilot study aimed to test key elements of a pilot RCT of the LLTTF online package. It demonstrated that a group of self-referring participants can be successfully recruited from the community and engaged in the RCT, with voluntary sector delivery. Several key methodological uncertainties in relation to study design and intervention delivery have been addressed, thus providing useful information for a future fully powered trial.

### Recruitment and Sample

First, recruitment was feasible, with 36 eligible individuals recruited within 3 phases of advertising in the Metro newspaper. The resources were limited in the current study, but it seems likely that, in a future funded RCT with an appropriate budget for UK-wide advertising, the required sample size (see the following section) could be achieved using a community-based recruitment approach. The community-based approach employed successfully identified those living with depression. The majority of participants had at least moderate symptoms of depression, yet less than one-third was currently under the care of their GP. This is in line with reports that there is a significant treatment gap with regard to depression [5]. Online interventions could serve as a potential treatment avenue to bridge the gap between onset of symptoms and access to psychological therapy.

### Intervention Delivery and Scalability

The completion and satisfaction rates in this pilot study demonstrate that it is feasible and acceptable to deliver the intervention in a community setting with voluntary sector support. Working alongside an established voluntary sector organization was a strength of our intervention, and the full RCT aims to broaden the number of organizations involved, including providing access more widely across the United Kingdom. This will test the ability to scale up and deliver to a higher volume of participants.

### Data Collection and Follow-up

It proved possible to record data, with a 78% follow-up rate at 3 months. However, only one-quarter of responding participants completed the economic analysis questionnaires at 3 months, indicating that the format (emailed rather than completed via SurveyMonkey) and length may need to be reconsidered, as it appears that this outcome measure was too burdensome.

### Power Calculation

Mood measures were collected at baseline and 3 months and used along with rates of follow-up to calculate the sample size for a future definitive RCT.

The primary analysis in the future substantive RCT will compare changes in PHQ-9 scores at 6 months between intervention groups. We will power the study to be able to detect a between-group difference of 5 points on the PHQ-9 score. A difference of 5 points is used to reflect a category change (from moderate to mild depression, for example) on the PHQ-9 and is clinically important.

Based on a 2-sample *t* test, a sample size of 36 participants per arm would be required to have 95% power. In the pilot, follow-up data were available for 78% at 3 months. The proposed study would have 1 follow-up assessment at 6 months, so we have accounted for a 25% drop-out rate for the full RCT.

### Limitations

A limitation of this study is that, although we determined that participants had significantly high mood scores upon entry into the study, a diagnostic interview was not used in the screening process. This approach was applied because some people prefer voluntary sector delivery so they can seek help without a diagnosis being made or creating a formal NHS record. However, it is acknowledged that defining the participants in terms of diagnosis would be useful for a research analysis. Therefore, in order to address this limitation while promoting participant choice, the future RCT could include an optional psychiatric interview or diagnostic assessment at baseline. This method was applied in a previous study in which 65% of participants agreed to take part in the diagnostic interview [19,21]. Negative impacts will be assessed in the full RCT using qualitative interviews including with those who drop out of the study.

### Conclusions

This pilot study, designed to test the trial design and the delivery of the online life skills LLTTF course, has helped inform the sample size calculation for a future RCT and determined that such a trial is feasible in relation to recruitment, delivery, and retention. Our results have addressed several methodological uncertainties and demonstrated the feasibility to conduct a definitive RCT within a community-based setting, delivered with support from nonspecialist voluntary sector support workers. The power calculation suggests a sample size of 72 is required for the full RCT. This pilot study strongly supports the potential to successfully recruit this required number of participants using a community-based recruitment strategy.

Delivery of the intervention by charity support workers was associated with reasonable participant satisfaction. However, efforts will need to be taken to improve completion rates in a future definitive RCT. Additionally, it proved problematic to conduct an economic analysis via emailed documents. It seems a telephone or other live interview might work more effectively for any future definitive RCT. Finally, in the future study, there would be advantages to collecting data at 6 months and possibly at 1 year to record long-term outcomes and whether any changes to mood and social function are sustainable.

## Appendix

Multimedia Appendix 1 CONSORT-eHEALTH checklist (V 1.6.1).

## Abbreviations

CBT: cognitive behavioral therapy
CSQ-8: Client Satisfaction Questionnaire-8
CSRI: Client Service Receipt Inventory
DA: delayed access
GAD-7: Generalized Anxiety Disorder-7
GP: general practitioner
IA: immediate access
iCBT: internet-based CBT
LLTTF: Living Life to the Full
mhGAP: mental health Gap Action Programme
NHS: National Health System
NICE: National Institute for Health and Clinical Excellence
PHQ-9: Patient Health Questionnaire-9
RCT: randomized controlled trial
WSAS: Work and Social Adjustment Scale
